# Machine learning enhanced optical spectroscopy for breast cancer diagnosis: A review

**DOI:** 10.1007/s10103-026-04882-9

**Published:** 2026-05-07

**Authors:** Mihir Nakul, Sanket Dinesh Rao, Manikanth Karnati, Farhath Aziz, Devadiga Pooja Bhaskar, Budheswar Dehury, Nirmal Mazumder

**Affiliations:** 1 Present Address: Department of Bioinformatics, Manipal School of Life Sciences, Manipal Academy of Higher Education, Manipal, India; 2 Department of Biophysics, Manipal School of Life Sciences, Manipal Academy of Higher Education, Manipal, India; 3 Osmania University, Hyderabad, India

**Keywords:** Spectrum analysis, Deep learning, Neural networks, Biomarkers, Optical spectroscopy

## Abstract

This literature review examines the transformative role of machine learning (ML) and deep learning (DL) in enhancing optical spectroscopy for breast cancer diagnosis. By synthesizing advancements from peer-reviewed studies (2015–2025), we evaluate how ML/DL integration improves the detection of malignancy-associated biochemical changes, enabling noninvasive, rapid, and accurate differentiation between healthy and cancerous tissues. This review highlights key spectroscopic modalities, such as Raman, fluorescence, diffusive optical spectroscopy (DOS), and photoacoustic spectroscopy (PAS), and their integration with AI-driven models, such as convolutional neural networks (CNNs), support vector machines (SVMs), and logistic regression. These techniques achieve diagnostic accuracies of up to 94% in subtype classification (e.g., luminal A, HER2-positive) by analyzing spectral biomarkers such as hemoglobin, lipids, and collagen. Challenges such as data variability, model interpretability, and clinical integration barriers are critically assessed. These findings underscore the potential of ML/DL-enhanced spectroscopy to standardize diagnostics, reduce unnecessary biopsies, and personalize treatment monitoring. Future directions emphasize the need for explainable AI (XAI), multimodal data fusion, and large-scale, diverse datasets to bridge translational gaps. By addressing technical, ethical, and regulatory hurdles, this integration promises to advance early detection, improve clinical outcomes, and reshape precision oncology.

**Graphical abstract:**

**Figure:**
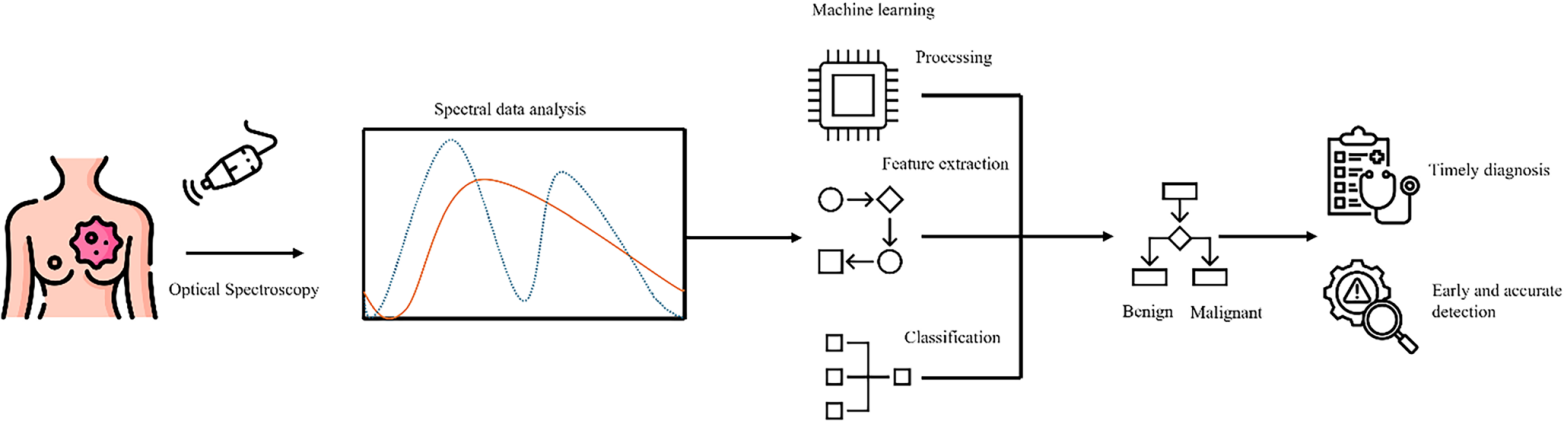
The graphical abstract illustrates the application of machine learning (ML) in optical spectroscopy for the diagnosis of breast cancer. ML enhances optical spectroscopy for breast cancer diagnosis through superior pattern recognition, early detection, and personalized treatment. It enables automation, high-throughput analysis, and multimodal data integration and improves diagnostic accuracy, consistency, and scalability.

## Introduction

Breast cancer involves the rapid multiplication of cells from breast tissues. The breast primarily consists of 2 tissues, glandular and stromal. Most breast cancers typically arise from glandular tissues, more specifically, the cells that line the milk ducts and lobules. Breast cancer accounts for approximately 11.6% of all cancer cases and is the second most diagnosed cancer among females [1–3]. Breast cancer predominantly affects women, with an increasing chance of occurrence with age. Approximately 80% of the cases are in women over the age of 50, and fewer than 5% of women are diagnosed before the age of 35 [4]. According to several studies, more than 685,000 deaths from breast cancer occurred worldwide in 2020 [2, 4, 5]. This number significantly decreased by the year 2021, with approximately 43,600 deaths out of 281,550 women who were diagnosed with the disease [1]. This decrease in the number of deaths over the years can be attributed to advanced treatment techniques and early diagnosis [2]. The World Health Organization (WHO) and IARC project significantly increased the burden of cancer, including breast cancer, which is expected to increase by more than 46% by 2040 [6]. The projected increase in the incidence of breast cancer worldwide, particularly in lower-resource settings, underscores the urgent need for strengthened prevention, early detection, and treatment strategies globally.

Although several factors influence the development and spread of cancer, genetic predisposition is a significant contributing factor. Mutations in the BRCA1 and BRCA2 genes increase the risk of cancer by 10% [7]. Breast cancers present themselves as lumps in the breast, 90% of which are benign in nature and painless [4]. Breast cancer can typically be identified as a change in the shape and size of the breast, dimpling, edema, blistering, changes in nipple morphology and discharge from the mammary glands, and in severe cases, ulceration can also be observed, which is why self-examination of the breasts from time to time is important [7, 8].

On the basis of the immunohistochemical receptors of certain hormones, such as estrogen receptor positive (ER+), progesterone receptor positive (PR+), human epidermal growth factor receptor positive (HER+) and triple negative, which lack the expression of any of the above receptors, breast cancer is classified into 4 subtypes: luminal A, luminal B, HER2 and TNBC [5]; Borunda et al., 2022). Knowing the subtype helps in better diagnosis and the development of an effective treatment plan. Early detection of breast cancer helps in treating approximately 90% of cases in the early stages and 50–80% in the middle and later stages [9]. The most common methods for the diagnosis of breast cancer include mammography, thermography and ultrasound [1, 4, 9]. Ultrasound provides a deep scan of the abnormality, whereas mammography focuses on the area of concern of the breast tissue, revealing benign or malignant abnormalities [1, 4]. On the other hand, thermography uses surface body temperature to generate thermal images, which are then converted to electrical signals [9]. While these systems categorize tumors on the basis of histopathology and receptor status, emerging molecular profiling reveals substantial heterogeneity within these subtypes, complicating diagnostic and therapeutic approaches [10]. Current screening modalities foundational to early detection face limitations in specificity (≤ 85%), patient comfort, and accessibility, particularly in resource-limited settings. Mammography’s reduced sensitivity in dense breast tissue (40–78%) and ultrasound operator dependency contribute to 15–30% false-negative rates across populations [11–13]. These gaps have spurred interest in nonionizing, cost-effective alternatives capable of detecting biochemical alterations preceding morphological changes.

In addition to the above diagnostic methods, several imaging techniques are employed for the diagnosis of breast cancer, including X-ray imaging, magnetic resonance imaging, magnetic resonance spectroscopy, and nonionizing imaging, such as optical imaging and spectroscopy [14]. Optical imaging and spectroscopy are recognized as real-time, highly sensitive, and noninvasive methods for detecting human cancers in hard-to-reach areas. These techniques utilize light propagation through tissue to evaluate its optical properties. Reflection, refraction, absorption and scattering are the main governing principles of light‒tissue interactions. Absorption is a process in which cells take in light molecules that are emitted from a light source, following the principles of Beer–Lambert’s law. Hemoglobin, a molecule that delivers oxygen to tissues, is the prime light absorber in the whole optical spectrum [14]. The total hemoglobin concentration reflects vascularization levels, making it a useful marker for monitoring angiogenesis in cancer progression [15]. A review by Chohan et al., [15] discussed several diverse biomarkers that serve as hallmarks in the detection of breast cancer. Biomolecules such as collagen, elastin, nicotinamide adenine dinucleotide (NADH), and flavin adenine dinucleotide (FAD) have been identified as key indicators in breast cancer diagnosis [15].

Optical spectroscopy techniques have been proven to exhibit the ability to mark the notable physical and chemical changes that take place in tissues and cells. This technique represents a breakthrough in the diagnosis of breast cancer because of its ability to differentiate healthy tissues from malignant tumors. Among the various spectroscopic methods, Raman spectroscopy (RS) is a fast, noninvasive, and effective diagnostic method. It operates on the principle of scattering, more specifically inelastic scattering. Hemoglobin, b-carotene, lipids and water act as the primary absorbers and scatterers of light [14]. Similarly, fluorescence spectroscopy is used to measure the concentration of biomolecules present in blood, urine or sputum, which function as biomarkers for detecting the presence and stage of cancer in the body [14]. While Raman and fluorescence spectroscopy continue to be used in diagnosis, recent advancements in the field have led to the development of more improved and efficient methods. Other spectroscopic methods that have significantly contributed to the diagnosis of breast cancer include diffusive optical spectroscopy (DOS) and photoacoustic spectroscopy (PAS). DOS uses near-infrared light to distinguish malignant and benign tissues by assessing various biomarkers, such as hemoglobin, water and lipids. PAS combines laser light and ultrasound to produce high-resolution images of the tumor vasculature and oxygenation, making it effective in detecting tumor hypoxia and monitoring treatment response in patients with breast cancer [16].

While the above diagnostic methods are widely used, several more approaches can be practiced on their own or combined with other methods for a more accurate diagnosis. Artificial intelligence, particularly machine learning (ML) and deep learning (DL), is a discipline of computer science that makes broad use of AI in the diagnosis of breast cancer [9]. Owing to recent advancements in technology, AI has gained remarkable recognition for its applications in medicine, particularly for the early diagnosis of diseases. Early detection via AI and machines, also known as computer-aided diagnosis (CAD), can be helpful in assisting radiologists in decreasing the mortality rate attributable to growing cancers [17]. Medical images are still mostly evaluated by trained radiologists, who can visually identify the presence or absence of disease, outline tumor boundaries, assess tumor responses to treatment, and detect disease relapses. These human skills serve as benchmarks against which AI and ML approaches are assessed. AI-based medical imaging is extensively carried out owing to its physical and virtual impact on medicine [18]. Medical images generated via machines are crucial for the appropriate diagnosis of disease. Machine learning (ML) is a subset of AI in which a machine is used to develop neural network-based algorithms, which can in turn help the machine understand and solve problems such as the human brain [18]. The key features of the ML model include adaptability, learning from past data and finding patterns, and making decisions for new datasets on the basis of previous patterns [17]. Deep learning (DL) is a subset of ML that is based on the concept of artificial neural networks (ANNs), which mimic the human brain in analyzing the characteristics that are required to identify images, detect diseases and process data [17, 18]. Deep learning (DL) begins by recognizing basic features in images, starting from the simplest elements. As it progresses through higher levels of learning, it builds upon these basic features to understand more intricate and complex patterns [9].

The following important questions are addressed in this review in order to give an organized summary of current developments in this field; Which optical spectroscopic methods are currently the most used for detecting breast cancer, and what biochemical data do they offer? In what ways do deep learning and machine learning techniques improve spectroscopic data analysis and diagnostic precision? What obstacles and restrictions now stand in the way of the extensive clinical application of AI-assisted optical spectroscopy? This paper attempts to give a thorough overview of the state of ML/DL-enabled optical spectroscopy today and its potential contribution to breast cancer diagnosis. By providing this comprehensive and critical overview, this review aims to provide readers with both a clear understanding of the current state and actionable insights for advancing the application of ML-enhanced optical spectroscopy in noninvasive breast cancer prognosis and diagnosis.

## Methodology

This narrative review synthesizes recent advances in the application of optical spectroscopy techniques combined with machine learning and deep learning approaches for breast cancer diagnosis. Although not a systematic review, a transparent and structured literature search strategy was adopted to ensure comprehensive coverage of relevant studies, minimize selection bias, and provide a rigorous, evidence-based synthesis of current developments, diagnostic capabilities, and remaining challenges in ML/DL-assisted spectroscopic cancer diagnostics.

### Literature search and selection

A thorough search of the literature was done using the databases PubMed, Scopus, Web of Science, and Google Scholar. To find other relevant studies, the reference lists of the retrieved publications and pertinent reviews were also examined. Peer-reviewed articles having experimental, methodological, or clinical studies on optical spectroscopy techniques including Raman spectroscopy, fluorescence spectroscopy, diffuse optical spectroscopy, and photoacoustic spectroscopy combined with machine learning or deep learning approaches for breast cancer diagnostics were given priority.

### Keywords used

Search strings combined the following terms in different Boolean combinations: “optical spectroscopy,” “Raman spectroscopy,” “fluorescence spectroscopy,” “diffuse optical spectroscopy,” “photoacoustic spectroscopy,” “biomedical spectroscopy,” “machine learning,” “deep learning,” “artificial intelligence,” “spectral analysis,” “convolutional neural networks,” “support vector machines,” “spectral biomarkers,” “breast cancer diagnosis,” “tumor classification,” and “optical imaging.”

### Time frame

Articles published between 2010 and 2025 were considered to capture both historical and recent advancements in breast cancer research.

### Language

Only studies published in English were included to ensure accurate interpretation and comparison of mechanistic findings.

### Inclusion criteria

- Original experimental, computational, or clinical studies investigating the use of optical spectroscopy techniques such as Raman spectroscopy, fluorescence spectroscopy, diffuse optical spectroscopy, or photoacoustic spectroscopy for breast cancer detection or characterization.
- Studies integrating machine learning or deep learning approaches (e.g., convolutional neural networks, support vector machines, random forest, or logistic regression) for spectral analysis, tumor classification, or biomarker identification.
- Articles reporting quantitative diagnostic outcomes, including performance metrics such as accuracy, sensitivity, specificity, or area under the curve (AUC) related to spectroscopic breast cancer detection.
- Review articles and methodological studies discussing advances in optical spectroscopy, artificial intelligence–assisted spectral analysis, or clinical translation of spectroscopic techniques were also consulted to provide contextual and comparative insights.

### Exclusion criteria

- Non-peer-reviewed literature, including conference abstracts without full papers, editorials, commentaries, or opinion pieces, were excluded to ensure scientific rigor.
- Studies focused on unrelated cancer types, non-optical diagnostic techniques, or imaging modalities not based on optical spectroscopy were excluded from the analysis.
- Reports lacking sufficient methodological detail, quantitative diagnostic outcomes, or clear relevance to machine learning–assisted spectroscopic analysis for breast cancer detection were not considered.
- Duplicate publications or overlapping reports from the same dataset were carefully screened and removed to avoid redundancy.

After applying the inclusion and exclusion criteria, a total of 121 peer-reviewed studies were selected for detailed analysis in this review.

## Types of optical spectroscopy

**Fig. 1:**
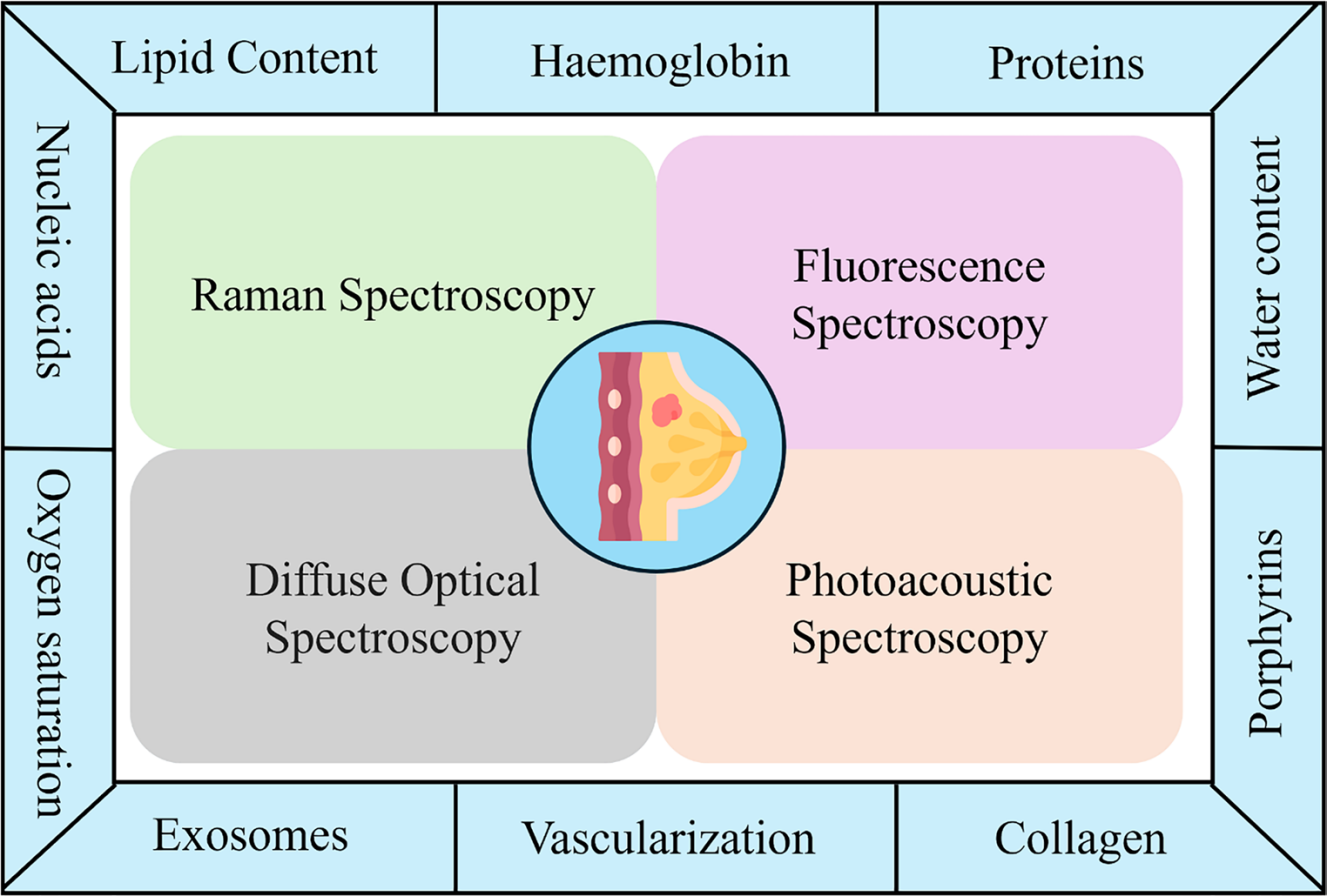
Schematic representation of various optical spectroscopy techniques:Raman, fluorescence, diffuse optical, and photoacoustic spectroscopy and their ability to detect key biochemical and structural biomarkers associated with breast cancer

Optical spectroscopy is a noninvasive technique that can sense minute variations and can provide quantitative biochemical information about the state of the tissue. Spectroscopy has the potential to differentiate between a malignant cancer-causing tissue and a benign noncancerous tissue. Additionally, it can detect specific optical biomarkers that are indicative of breast cancer progression (Fig. 1). A systematic comparison of optical spectroscopy techniques highlights that each modality provides complementary diagnostic information as shown in Table 1.

**Table 1:** Comparision of the various optical spectroscopic modalities and their strengths and limitation

Technique	Key Detectable Biomarkers	Information	Major Strengths	Limitations	Reference
Raman Spectroscopy (RS)	DNA/RNA, proteins, lipids, amino acids, collagen, carotenoids	Label-free detection of vibrational bands Limit of detection ~ ng/mL or ppm Spectral resolution ~ 40 μm	High molecular specificity; capable of identifying biochemical alterations and tumor subtypes	Weak signal intensity; longer acquisition time; limited depth penetration	Shipp et al., [19] Short et al., [20]
Fluorescence Spectroscopy (FS)	NADH, FAD, miRNAs, HER2 proteins, circulating tumor DNA, exosomes	Rapid detection of fluorescence and auto-fluorescence intensities Limit of detection ~ ng/mL or ppm	High sensitivity; rapid detection; strong signals for metabolic biomarkers; suitable for real-time monitoring	Requires fluorescent probes or endogenous fluorophores; photobleaching; limited depth	Melanthota et al., [21] Maciel et al., [22]
Diffuse Optical Spectroscopy (DOS)	Oxyhemoglobin (HbO₂), deoxyhemoglobin (HHb), total hemoglobin, oxygen saturation, water, lipids, collagen	Label-free quantification of tissue chromophores Tissue penetration depth ~ 1–5 cm (near-infrared range 650–1000 nm) Spatial resolution ~ 5–10 mm (depending on source–detector separation)	Noninvasive functional imaging; deeper penetration; quantitative measurement of tissue physiology	Lower spatial resolution; complex inverse modeling required	Tromberg et al., [23] Durduran et al., [24]
Photoacoustic Spectroscopy (PAS)	Hemoglobin, oxygen saturation (sO₂), lipids, water, collagen, tryptophan	Level of detection ~ 100 ppm	High spatial resolution with deep penetration; strong contrast for vascular structures; functional and structural imaging	Requires specialized instrumentation; signal processing complexity	Saalberg et al., [25] Priya et al., [26]

### Raman spectroscopy (RS)

**Fig. 2:**
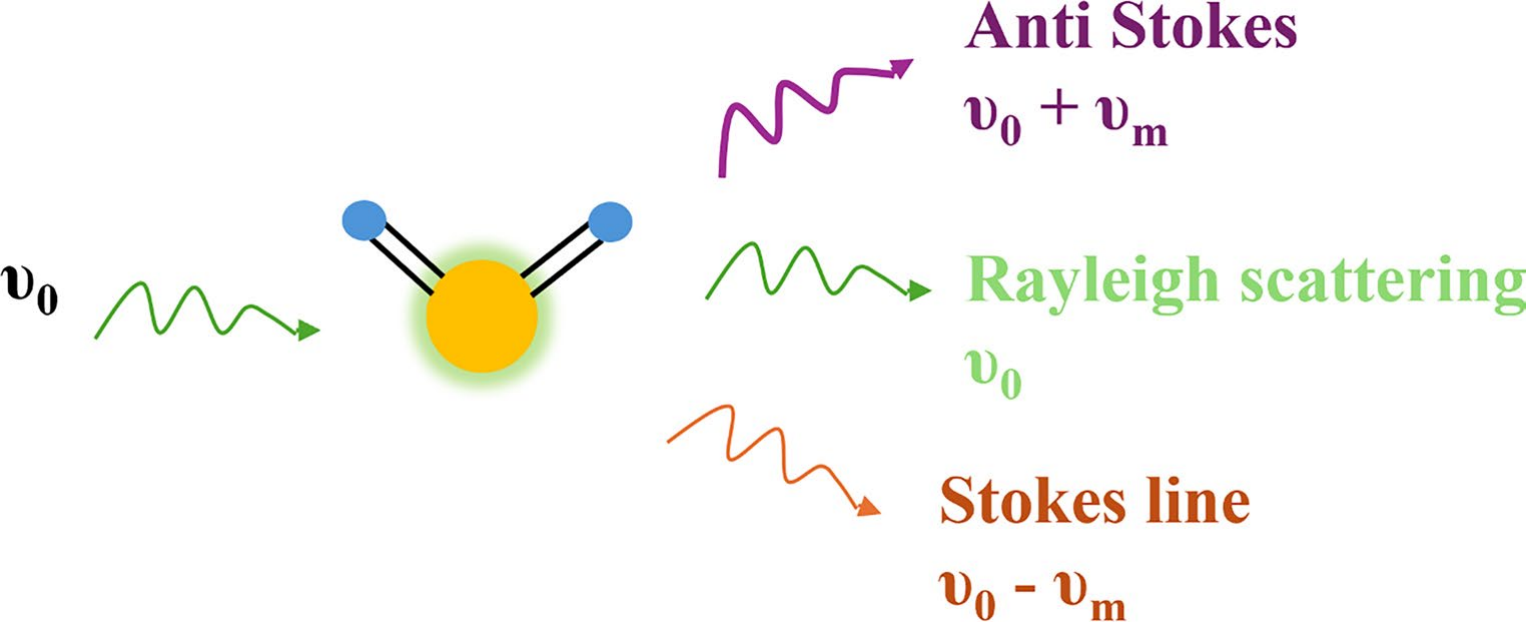
Schematic illustration of light scattering via Raman spectroscopy. Rayleigh scattering involves no energy change, Stokes scattering results in energy loss, and anti-Stokes scattering involves energy gain by the photon

Raman spectroscopy is a powerful type of molecular vibrational spectroscopy used to detect molecular vibrations and provides information about a sample’s composition [27]. It provides a fingerprint of vibrational spectra, which identifies each component of the sample [12, 28]. Raman spectroscopy is based on the principle of inelastic scattering of monochromatic light, which interacts with molecules; most light is scattered elastically (Rayleigh scattering), and a small fraction undergoes energy exchange with molecular vibration, which is known as Raman scattering. If a molecule gains energy, it results in Stokes scattering, and if it loses energy, it results in anti-Stokes scattering. The Raman shift reveals the vibrational energy levels unique to each molecule (Fig. 2 [29, 30]. Raman spectroscopy distinguishes four major subtypes of breast cancer: luminal A, luminal B, HER-2+, and TNBC. In Raman spectroscopy, unique spectral features can be observed, which are associated with DNA, lipids, proteins, and amino acids, which act as biomarkers for cancer detection [31, 32].

This can be accomplished by directly analyzing tissue where we observe alterations in DNA/RNA, lipid, and protein profiles in tumors. For example, a reduction in lipid content and an increase in nucleic acid can signal proliferation [33], or liquid biopsy analysis involves the collection and analysis of tumor-derived material from body fluids such as blood and plasma. The key components detected are exosomes, circulating tumor cells (CTCs), cell-free DNA/RNA, and proteins [32, 34–37].

Proteins are central to cancer biology, as they influence apoptosis, proliferation, cell signaling, and immune invasion, which are the main reasons for cancer development. In Raman spectroscopy, various types of proteins, such as phenylalanine (1002 cm⁻¹), which is elevated in tumor tissue, are detected at different wavenumbers, indicating an increase in protein synthesis [29, 38]. Amide III (~ 1271 cm⁻¹) is associated with secondary structures [39] and aromatic rings (1518 cm⁻¹), where HER2 + tumors show a decrease in intensity, which reflects a reduced aromatic content [29, 35, 40]. Lipids are the second biomarker that contributes to membrane structure and cellular signaling, and any alteration in lipid metabolism can act as a hallmark for cancer. For example, CH2 bends (~ 1448 cm⁻¹), and a reduction in CH2 in tumor tissue indicates membrane degradation or lipid depletion [32, 38, 39]. While CH3/CH2 (~ 1309 cm⁻¹) reflects fatty acid chain content, which is low in aggressive tumors, CH stretching vibrations (2850–2950 cm⁻¹) may be reduced in cancer serum due to metabolic changes [38, 39].

Nucleic acids such as DNA and RNA act as biomarkers that reflect tumor cell replication and transcriptional activity in the wavenumber range of 724,754 cm⁻¹ ring breathing modes of nucleotides elevated in cancer tissues, 800–900 cm⁻¹ backbone phosphate vibration increases in cancer, and 941 cm⁻¹ DNA‒protein interaction is reduced in HER@-cancer [12, 32, 35, 39]. Alterations in collagen reflect tumor-associated extracellular matrix remodeling at a wavenumber range of 869 cm⁻¹ (proline and hydroxyproline) reduction in cancer due to collagen degradation [38, 40]. Exosomal biomarkers are extracellular vesicles that carry molecular signatures of parent cells and include HER2 and EGFR, which are tracked dynamically during therapy to predict the response. CTCs serve as indicators of metastasis and disease function where EpCAM and HER2 are detected via SERS-conjugated antibodies, which allows noninvasive monitoring, and microcalcifications are early markers of carcinoma in situ where phosphate vibrations specific to malignancies are detected via Raman spectroscopy [34, 35, 41].

### Fluorescence spectroscopy

**Fig. 3:**
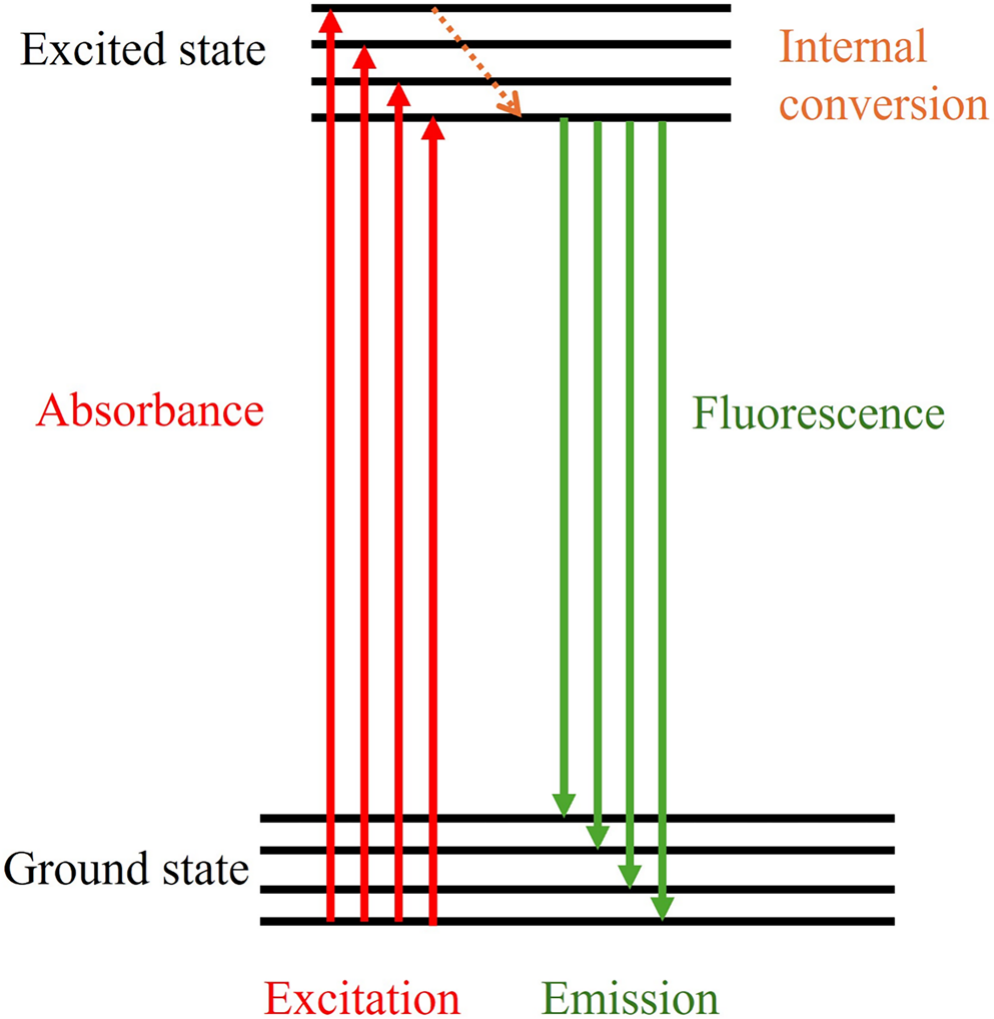
Schematic illustration showing the Jablonski diagram of fluorescence: Molecules absorb light and convert to higher electronic states (excitation), where they undergo internal conversion to the lowest vibrational level of the excited state before returning to the ground state via fluorescence

Fluorescence spectroscopy is a widely used analytical tool for detecting specific biomarkers in breast cancer. These biomarkers include proteins, nucleic acids, and extracellular vehicles such as exosomes [42]. These methods are based on certain factors, such as fluorescent probes, quantum dots, and nanomaterials, to increase signal sensitivity and specificity [42–44]. Fluorescence is a photophysical process in which a substance absorbs light at a shorter wavelength before emitting light at a longer wavelength (Fig. 3). When a molecule absorbs photons, its electrons are stimulated to higher energy levels. As electrons return to their ground state, they emit energy in the form of visible or near-visible light. In breast cancer diagnostics, fluorescence spectroscopy provides molecular contrast by detecting emitted light and excited wavelengths from the fluorophore. This fluorophore interacts with some specific biomolecules (biomarkers), leading to measurable fluorescence intensity [45]. This helps us to detect cancerous cells vs. normal breast cells.

Insight into tumor metabolism and redox states is provided by the reduced forms of nicotinamide adenine dinucleotide (NAD) and flavin adenine dinucleotide (FAD), which act as markers of metabolic activity in cancer cells and can be used to evaluate how estrogen receptor expression affects metabolic processes [46]. As there is an increase in NADH/FAD, a metabolic shift can be observed due to increased glycolytic activity (Warburg effect) and mitochondrial dysfunction in tumors, which leads to alterations in redox states and high fluorescence emission [42, 47]. Exomes derived from tumors carry oncogenic miRNAs, e.g., miR-21, mutated DNA (ctDNA), and tumor-specific proteins such as HER2 and ER. All of these compounds can be extracted from blood plasma, urine, or saliva via ultracentrifugation, size-exclusion chromatography, or immunoaffinity capture [44, 45, 48–51].

A fluorescence spectrometer records the intensity and calibrates a curve, determining the exact miR-21 concentration. A biological sample containing miR-21 binds to a complementary ssDNA probe attached to fluorescent carbon dots (CDs) and converts to a ds-hybrid [49, 52]. This leads to a decrease in fluorescence intensity, indicating an elevated level of miR-21, which is associated with cancer progression [49]. If mutated ctDNA is present, the fluorescent probe binds to it and enhances the fluorescence intensity, confirming that a mutation is present. Moreover, excitation light (e.g., 488 nm) is used to excite the fluorophore, which emits fluorescence [48]. While HER2 promotes cell growth and is overexpressed in aggressive breast cancer, ER binds estrogen and influences the development of hormone-dependent breast cancer. This leads to changes in fluorescence intensity [44, 45]. Overall, fluorescence spectroscopy is a valuable tool that provides insight into tissue metabolism, composition, and therapeutic response, making it a promising tool for breast cancer detection.

### Diffusive optical spectroscopy

**Fig. 4:**
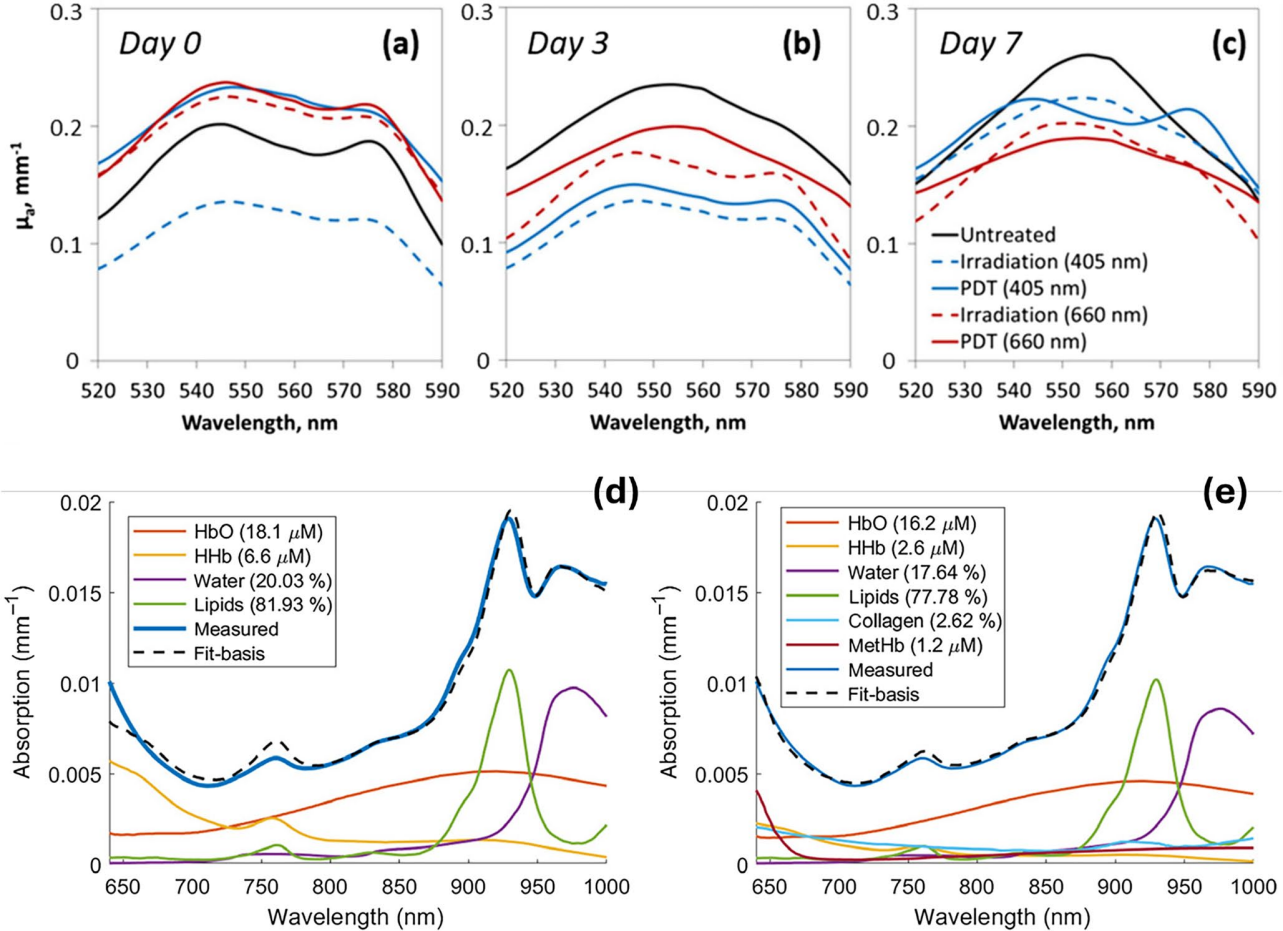
(**a**–**c**) Reconstructed absorption spectra of tumor tissue on Day 0 and after PDT on Days 3 and 7 using 405 nm and 660 nm irradiation, showing changes in the hemoglobin absorption peaks. Reproduced from Orlova et al., [55], Photonics 9, 19 (2022), under the terms of the Creative Commons Attribution 4.0 International License (CC BY 4.0). (**d**–**e**) Quantitative decomposition of measured absorption spectra into individual tissue chromophores, including oxygenated hemoglobin (HbO), deoxygenated hemoglobin (HHb), water, lipids, collagen, and methemoglobin (MetHb). Reproduced from Vasudevan et al., [54], J. Biomed. Opt. 26(6), 065004 (2021), under CC BY 4.0

Diffusive optical spectroscopy (DOS) is a noninvasive technique that uses near infrared (NIR) light to probe tissue physiology. In this technique, NIR light interacts with turbid biological tissues where multiple scattering dominates over absorption, resulting in a diffusely scattered photon field. The transport of light through such media is effectively described via diffusion theory [53]. In breast cancer, DOS is used to obtain spectroscopic information about optical biomarkers such as the hemoglobin concentration, oxygen saturation, water and lipid contents, vascularisation, collagen deposition and scattering properties [14]. Collagen and methemoglobin (metHb) are potential absolute biomarkers for breast cancer. metHb has lower interpatient variability, making it a more reliable marker of malignancy, and collagen shows greater variability since it is also present in normal breast tissue. Vasudevan et al., [54] described metHb as a promising discriminator of malignant and benign lesions (Fig. 4d-e). The chromophore concentrations were calculated from the tissue absorbers, and their absorption spectra were fitted into known molar extinction coefficient spectra via ordinary least squares regression. The region of interest (ROI) was defined on both the lesion-containing breast and the contralateral normal breast, and the lesion-to-normal ratio (L/N) was used to differentiate benign from malignant tumors [54].

Tissue oxygenation is a very important cancer hallmark, and the DOS effectively measures the concentrations of oxyhaemoglobin (HbO2) and deoxyhaemoglobin (HHb), allowing the assessment of tissue blood oxygen saturation (StO₂) and total haemoglobin (tHb) as a measure of blood content.Orlova et al., [55] demonstrated the feasibility of monitoring photodynamic therapy (PDT) responses via DOS before and after PDT with red (660 nm) and blue (405 nm) light. Before treatment, all the tumor groups had similar oxygen saturation (StO₂) levels, with prominent oxyhemoglobin peaks (540 & 570 nm). Posttreatment, red-light PDT led to a rapid decrease in StO₂, whereas blue-light PDT maintained higher oxygen levels, suggesting that blue-light PDT (405 nm) better preserves tumor oxygenation, which may impact therapeutic effectiveness (Fig. 4: a-c). DOS measurements confirmed that the chlorin e6 photosensitizer did not interfere with the spectral readings [55].

A tumor’s high-water content indicates edema and enhanced cellularity. Compared with benign tissue, malignant breast lesions have higher water contents and lower lipid contents. The Hb concentration increased when the skin-to-chest wall distance decreased from approximately 20 mm. As the distance decreased from approximately 15 mm, the water content increased, whereas the lipid content decreased. The combination of DOS with ultrasonography improves accuracy in evaluating breast structure and detecting abnormalities [56]. In another study by Ohmae et al., [57], the lipid and water contents of TD-DOS were determined via tissue absorption owing to vibrational overtones of lipid C‒H bonds (930 nm) and water O‒H bonds (978 nm) [57]. DOS has emerged as a powerful, noninvasive tool for studying the optical and physiological properties of breast tissue to analyze important biomarkers and its ability to distinguish between malignant and benign lesions, monitor treatment responses, and supplement imaging modalities.

### Photoacoustic Spectroscopy

**Fig. 5:**
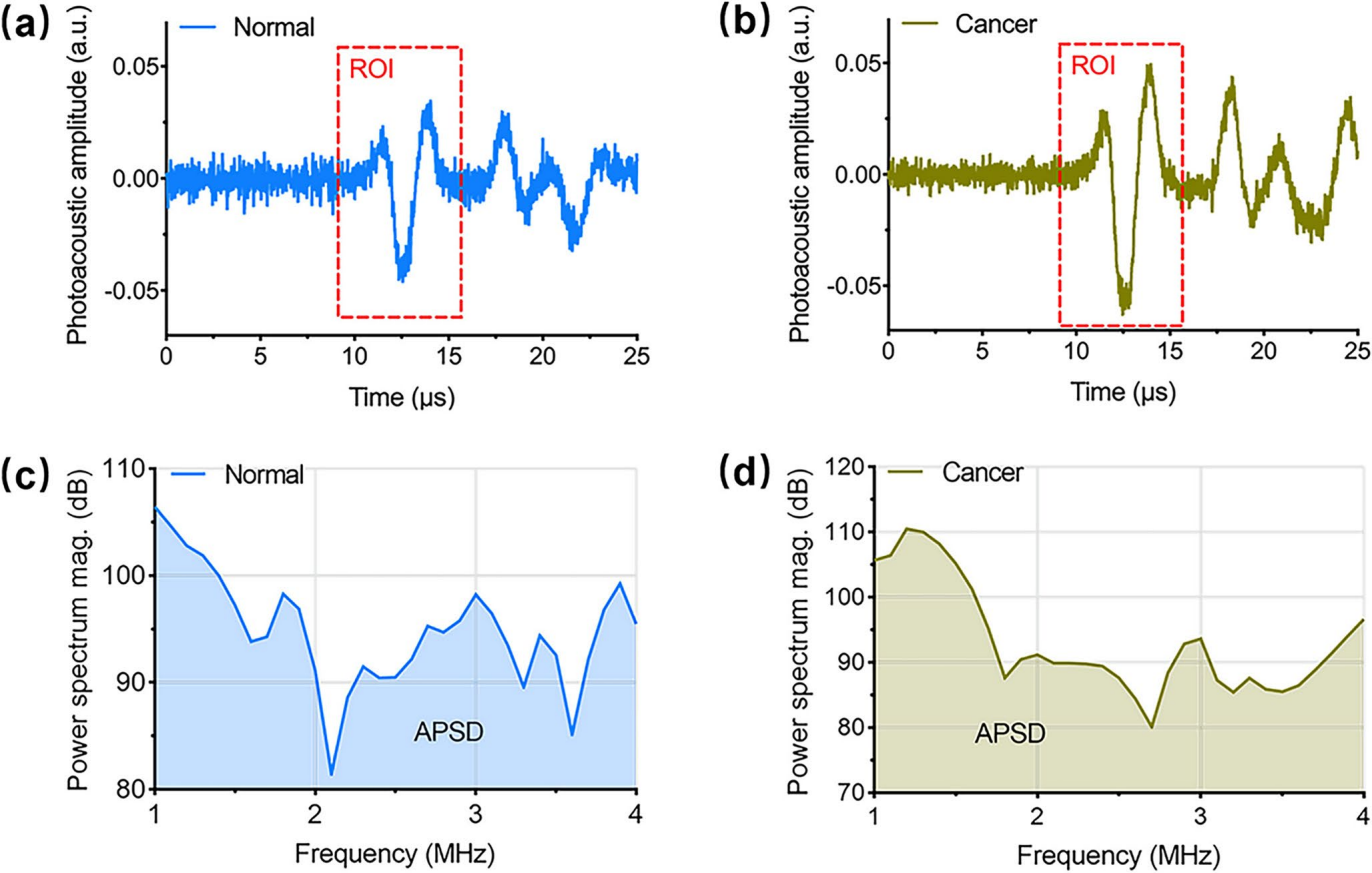
Acquisition of the photoacoustic spectrum involves capturing representative in vivo photoacoustic signals from (**a**) normal and (**b**) cancerous tissue samples. Additionally, power spectral representations are shown for (c) normal and (d) cancerous tissues. Reproduced from Li et al., [59], Journal of Biophotonics, 2024; 17: e202400371, under the terms of the Creative Commons Attribution (CC BY 4.0) license

Photoacoustic spectroscopy (PAS) is a technique that combines optical absorption and acoustic detection to analyze the composition and properties of materials. In breast cancer, the PAS is used to obtain spectroscopic information about optical biomarkers such as water and lipid contents, vascularisation, and amino acid and collagen deposition in the tumor microenvironment. Collagen, a major component of the extracellular matrix (ECM), undergoes significant remodeling during tumor progression. This remodeling supports cancer invasion and metastasis. Traditional methods (histopathology, second-harmonic generation, and mass spectrometry) are invasive and mostly limited to ex vivo analysis, making them unsuitable for early-stage breast cancer screening. Li et al., [58] used PAS laser wavelengths ranging from 1200 to 1700 nm. Their study introduced the area of power spectrum density (APSD) as a novel PA parameter to semiquantify collagen levels. The acinar tissues presented lower APSD values in the collagen-dominated wavebands, confirming the occurrence of collagen degradation in the tumors (Fig. 5). Additionally, greater variation in APSD values among cancerous samples suggests extracellular matrix (ECM) heterogeneity, providing noninvasive biochemical insights into carcinogenesis [59]. Cancerous tissues presented lower APSD values in the collagen-dominated wavebands (1260–1370 nm), confirming the occurrence of collagen degradation in the HER2 and TNBC subtypes. Further spectral analysis revealed three key biomacromolecule-dominated optical absorption bands, namely, the collagen (1260–1370 nm), lipid (1200–1250 nm & 1510–1700 nm), and water (1380–1500 nm) bands, and effectively differentiated the luminal (high collagen) from the HER2 and TNBC (low collagen) subtypes [58].

Tryptophan, an essential amino acid, plays a crucial role in cancer metabolism. Tumor cells take up tryptophan and metabolize it through the kynurenine pathway, which promotes immunosuppression and tumor progression. Rodrigues et al., [60] demonstrated that serum tryptophan levels decrease as tumors grow, suggesting that tumors actively take up tryptophan. Tumor tissues were excited with 281 nm pulsed laser light, a wavelength specifically absorbed by tryptophan. Tumor tissues presented increased PAS signal intensity in tryptophan-dominated spectral regions. As the tumors progressed (5, 10, 15, and 20 days after MCF-7 injection), tryptophan-related PAS signals increased, confirming their accumulation in cancerous tissues. Serum tryptophan levels decreased, demonstrating that tumors actively metabolize this biomarker [60, 61]. Tumor tissues, including those in breast cancer, are often characterized by abnormal vasculature and hypoxic regions due to rapid cell proliferation and an inefficient blood supply. These hypoxic conditions not only promote tumor progression and metastasis but also contribute to resistance to therapies. PAS enables this functional assessment by utilizing modulated or pulsed laser light to induce localized thermoelastic expansion in hemoglobin-rich blood vessels. The subsequent generation of acoustic waves, which differ depending on the light absorption properties of oxyhemoglobin (HbO₂) and deoxyhemoglobin (Hb), allows for the determination of oxygen saturation levels. Multiwavelength PAS, particularly when laser wavelengths such as 680 nm and 808 nm are used, exploits the distinct absorption peaks of HbO₂ and Hb to quantify sO₂ in tissue regions. This capability is especially valuable in breast cancer diagnostics, where hypoxia is a hallmark feature of aggressive tumor regions. A wavelength-modulated differential PAS (WM-DPAS) improves detection sensitivity by using two modulated wavelengths to suppress background noise and amplify the signal difference between oxygenated and deoxygenated hemoglobin [43]. PAS enables detailed spectral analysis of key biomarkers, offering insights into the tumor microenvironment, subtype classification, vascularization, and hypoxia. Its ability to provide molecular specificity with deep tissue resolution makes the PAS a powerful tool for early detection, monitoring progression, and evaluating therapeutic responses in breast cancer diagnostics.

## Machine learning and deep learning

The fields of ML and DL have completely revolutionized data-driven decision-making across various disciplines, including medical diagnostics. As optical spectroscopy continues to demonstrate promise in noninvasive breast cancer diagnostics, its integration with ML and DL has become increasingly significant. These data-driven approaches enable automated pattern recognition, feature extraction, and predictive modeling, making them highly suitable for analyzing complex spectral data from optical spectroscopy [62]. This section introduces the foundational concepts of artificial intelligence (AI), ML and DL, followed by a discussion of their suitability for analyzing optical spectroscopy data in breast cancer applications. The relationship between AI, ML, DL, and ANNs can be understood hierarchically, where DL represents a subset of ML, which itself is a subset of AI. AI refers to the overarching concept of developing machines that can replicate human behavior and execute functions typically associated with human intelligence. It employs diverse methodologies to allow machines to demonstrate intelligent behavior by emulating human cognitive functions, including reasoning, perception, problem-solving, and learning. ML, a branch within the broader field of AI, empowers computers to learn from data and make informed decisions without being explicitly programmed [63]. ML techniques are typically categorized into four major types: supervised learning, unsupervised learning, semisupervised learning, and reinforcement learning [62]. Supervised learning involves training algorithms on labeled datasets, allowing models to discern input‒output relationships and generate predictions. Frequently used algorithms in this category include support vector machines (SVMs), random forests (RFs), and k-nearest neighbors (k-NNs), which are particularly effective for classification and regression tasks in spectroscopy-based diagnostic applications. In contrast, unsupervised learning focuses on uncovering hidden patterns and structures within unlabeled data. Approaches such as k-means clustering and principal component analysis (PCA) are instrumental in dimensionality reduction and the categorization of spectral features [50]. Semisupervised learning represents a blend of supervised and unsupervised techniques that leverage both labeled and unlabeled data. This approach is especially useful in optical spectroscopy when large volumes of spectral data lack labels and only a small fraction is annotated. By utilizing information from both sources, models can be trained to recognize chemical signatures or interpret complex spectral data, which would otherwise be costly and labor intensive to label manually [64]. Reinforcement learning involves training models through interactions with an environment where they learn optimal strategies by receiving feedback in the form of rewards or penalties. Although it has not yet been widely applied in spectroscopy, reinforcement learning shows promise for enhancing the efficiency of diagnostic decision-making systems [58].

DL, a distinct subset within ML, employs artificial neural networks composed of multiple layers to capture intricate patterns within large-scale datasets. Unlike conventional ML techniques, which typically rely on manual feature extraction, DL algorithms are capable of autonomously learning hierarchical features directly from raw data. This ability makes DL especially well-suited for analyzing high-dimensional and nonlinear spectral data [65]. This characteristic makes DL particularly valuable for spectroscopic applications where high-dimensional, nonlinear data are common. The field of DL has expanded rapidly across numerous domains, with specialized implementations demonstrating exceptional performance in fields ranging from computer vision to spectroscopic analysis. As noted in comprehensive reviews, DL models have completely changed the concept of AI by enabling more sophisticated pattern recognition and feature extraction without manual intervention [66, 67].

Artificial neural networks (ANNs) consist of multiple layers of interconnected nodes (or neurons) that process input data via weighted links. Serving as the backbone of DL, ANNs are capable of approximating complex, nonlinear functions. However, these methods are often computationally demanding and typically require large datasets to perform effectively [68]. Convolutional neural networks (CNNs), which were originally designed for image analysis, have shown marked effectiveness in processing spectral and spectral imaging data. By using convolutional filters, CNNs can identify localized features in spectra such as shifts or peaks that may correspond to specific biomarkers [69, 70]. Recurrent neural networks (RNNs), along with their advanced variants, such as long short-term memory (LSTM) and gated recurrent units (GRUs), are well suited for handling sequential data and capturing temporal dependencies. While not yet widely adopted in optical spectroscopy, these architectures hold promise for analyzing time-resolved or dynamic spectral information [71]. Transfer learning enables the reuse of models pretrained on related tasks, thereby reducing the need for extensive labeled datasets. This approach is particularly advantageous in medical spectroscopy, where data collection can be both costly and labor intensive. Generative models such as variational autoencoders (VAEs) and generative adversarial networks (GANs) are employed to generate synthetic spectral data, augment existing datasets, and improve model generalizability. These models also support tasks such as anomaly detection and unsupervised feature extraction, contributing to more robust and flexible analytical pipelines [72].

The inherent complexity of optical spectroscopy data, marked by high dimensionality, subtle spectral variations, and potential measurement artifacts, poses considerable challenges for conventional statistical methods [73, 74]. Traditional analytical techniques often lack the precision and adaptability needed to derive clinically meaningful insights from such intricate datasets. However, recent developments in ML and DL have shown substantial promise in addressing these limitations [75]. ML and DL methods are particularly well equipped to handle the high-dimensional and nonlinear nature of spectral data. These algorithms can effectively model complex relationships while minimizing the risk of overfitting, especially when integrated with dimensionality reduction strategies such as PCA or autoencoders [76–78]. These techniques preserve essential variance in the data while reducing noise and redundancy. Furthermore, deep neural networks excel at automatic feature extraction, enabling the detection of spectral signatures linked to biochemical changes in malignant tissues (Warrier et al., 2022; [75, 79]). This ability to uncover subtle spectral distinctions, often beyond the reach of traditional analyses or expert interpretation, is critical for improving diagnostic sensitivity. Empirical studies highlight the enhanced accuracy and generalizability of DL models, particularly when trained on diverse and augmented datasets. Model performance is further refined through practices such as cross-validation, dropout regularization, and ensemble approaches [79–81]. Crucially, the scalability of these models supports their application across a range of spectroscopic techniques, including Raman, fluorescence, and diffuse reflectance spectroscopy and across various clinical settings [78, 82]. This versatility underscores their potential for broader translational implementation and seamless integration into diagnostic workflows. Collectively, the ML and DL approaches represent powerful tools for advancing optical spectroscopy-based diagnostics. The ability of these methods to handle complex data structures, uncover diagnostically relevant patterns, and provide scalable, interpretable outputs underscores their potential role in next-generation cancer detection platforms.

The selection of a ML or DL model is not arbitrary but is fundamentally dictated by the physical and mathematical characteristics of the spectroscopic signal. Understanding this relationship is critical to avoid application without knowing how it works and to ensure model reliability. Raman spectra are characterized by thousands of features such as wavenumbers and a low signal-to-noise ratio (SNR). ID-CNNs are uniquely suited for this data because their convolutional filters act as automated feature extractors that can remove noise from the signal and identify narrow vibrational peaks without manual preprocessing. DOS typically captures broad physiological features like hemoglobin and water content across a limited number of wavelengths. Because this data represents well-understood biological markers, linear models like Logistic Regression or LDA are often preferred; they offer high interpretability and are less prone to overfitting on the smaller patient cohorts common in clinical DOS studies. PAS generates acoustic pressure waves that are time-resolved. Analyzing these signals requires models capable of handling temporal dependencies and nonlinearities. SVMs with RBF kernels or ResNets with Attention Mechanisms excel here by transforming time-frequency representations via Wavelet transforms into robust classification features.

## Application of ML/DL in optical spectroscopy for breast cancer

This part of the review examines the integration of various spectroscopic techniques with ML approaches for cancer detection, classification, and biomarker discovery, with a particular focus on breast cancer diagnostics. We discuss current research on Raman spectroscopy, photoacoustic spectroscopy, fluorescence spectroscopy, and diffuse optical spectroscopy, highlighting both their individual contributions and the overarching trends and challenges in the field.

### Raman spectroscopy with convolutional neural networks

**Fig. 6:**
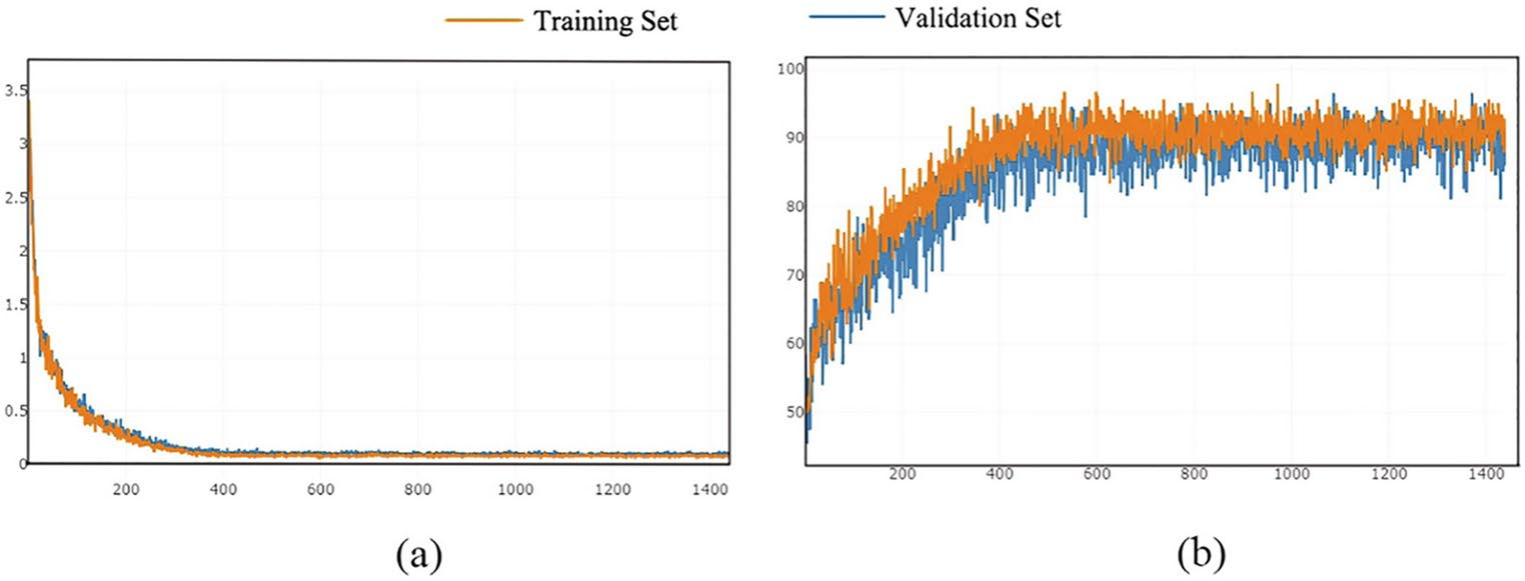
The loss function curves (**a**) and the accuracy curves (**b**) of 1D-CNN model to visulaize the training in real time, the figure adopted from Ma et al., [84]

Raman spectroscopy has gained prominence as a valuable analytical tool for breast cancer diagnosis, enabling rapid and noninvasive examination of biological samples. By measuring the vibrational and rotational energy modes of molecular functional groups, this technique generates unique spectroscopic signatures that reflect the underlying biomolecular composition [83]. When applied for breast cancer detection, Raman spectroscopy is effective at identifying molecular and structural variations between healthy and malignant tissues, capturing differences across critical biomolecules such as nucleic acids, proteins, lipids, and carbohydrates [84]. The spectral differences between healthy and cancerous breast tissues are significant and diagnostically valuable. For example, studies have reported decreased intensities of specific Raman bands (1298, 1437, 1742 cm⁻¹) in cancerous tissues, indicating a lower lipid content due to increased energy consumption during cancer cell proliferation. Similarly, shifts in the Raman bands at 865 cm and 1116 cm⁻¹ suggest important conformational changes in proteins and nucleic acids associated with malignancy [84]. Ma et al., [84] pioneered the application of a one-dimensional CNN (1D-CNN) for breast cancer tissue classification via Raman spectroscopy (as shown in Fig. 6). Their model architecture incorporated a convolutional layer, a pooling layer, and two fully connected layers with batch normalization, allowing for automatic feature extraction directly from raw spectral inputs and eliminating the need for manual feature engineering. The model demonstrated strong diagnostic performance, achieving 92% accuracy, with a sensitivity of 98% and specificity of 86% in distinguishing between healthy and cancerous breast tissues. Zeng et al., [85] compared three DL models for classifying triple-negative and HER2-positive breast cancers: the neural network language model (NNLM), bidirectional long short-term memory network (BILSTM), and CNN. Among these, the CNN model outperformed the others, achieving an accuracy of 91.11%, compared with 87.78% for the NNLM and 90.37% for the BILSTM. Their CNN architecture included a convolutional layer, a max pooling layer, a flattening layer, and a fully connected layer, along with batch normalization, to increase training efficiency and model convergence. In another study, Li et al., [83] introduced an optimized CNN model for breast cancer subtype classification via the Sparrow search algorithm (SSA). This optimization focused on tuning critical CNN hyperparameters such as the number and size of the filters, the learning rate, and the batch size. The SSA-enhanced CNN achieved a classification accuracy of 95.34% (± 2.18%) across five breast cancer subtypes—luminal A, luminal B, claudin-low, basal, and HER2-positive—demonstrating a significant performance advantage over traditional machine learning techniques.

Recent investigations into the application of convolutional neural networks (CNNs) in Raman spectroscopy for breast cancer diagnosis have leveraged diverse biological sample types. Serum samples, which offer a minimally invasive and reproducible method for disease detection, have been widely used. For example, Wang et al., [86] collected serum from 241 healthy volunteers, 463 breast cancer patients, and 100 ductal carcinoma in situ (DCIS) patients. Their CNN model achieved a remarkable classification accuracy of 98.76%. Similarly, Zeng et al., [85] used serum samples from 23 triple-negative breast cancer patients, 22 HER2-positive patients, and 30 healthy controls. Serum analysis is noninvasive, cost-effective, and highly reproducible. Fresh breast tissue samples enable direct analysis of molecular changes within the tumor microenvironment. Ma et al., [84] collected tissue samples from 20 patients, with each patient providing both healthy and cancerous breast tissue specimens. This paired-sample approach effectively reduced individual differences that might confound the classification results. Additionally, J. Li et al., [83] utilized established breast cancer cell lines—MCF-7, BT474, MDA-MB-231, MDA-MB-468, and SKBR3—and normal breast epithelial cells (Hs 578Bst) to develop and validate their classification model under highly controlled experimental conditions. Cell lines offer highly controlled experimental conditions for spectroscopic analysis. Across multiple studies, CNNs have consistently outperformed traditional ML approaches for Raman spectral analysis. Wang et al., [86] compared CNNs with support vector machines (SVM), random forest (RF), and K-nearest neighbor (KNN) methods for DCIS and breast cancer classification. The CNN achieved 98.76% accuracy, which was significantly higher than those of the SVM (94.63%), RF (80.99%), and KNN (78.93%) methods. J. Li et al., [83] demonstrated that their CNN model (95.34% ±2.18% accuracy) outperformed SVM (94.90% ±1.88%), PLS-DA (94.52% ±2.22%), and KNN (80.00% ±5.27%) for breast cancer subtype classification. Ma et al., [84] reported that the 1D-CNN outperformed both the Fisher discrimination analysis (FDA) and the SVM for breast tissue classification, with at least 3.5% higher overall accuracy.

The superior performance of CNN models stems from several key advantages, such as automatic feature extraction from raw spectral data without manual intervention, better handling of nonlinear patterns in complex spectral data, a reduced need for extensive preprocessing and feature selection, a stronger generalization ability with a lower risk of overfitting and enhanced stability through data augmentation techniques. A significant advancement in CNN applications for Raman spectroscopy is the development of visualization techniques to identify critical spectral features.Li et al., [83] applied gradient-weighted class activation mapping (Grad-CAM) to visualize the regions in Raman spectra that contributed most significantly to classification decisions. This approach computes the importance of each feature map by performing global average pooling on the gradients of the last convolutional layer, thereby enabling visualization of the spectral regions that most strongly influence the model’s classification decisions. The critical features identified through Grad-CAM matched well with established immunohistochemistry information, validating the biological relevance of the identified spectral regions. Studies have investigated whether full spectra or specific spectral regions contribute the most to classification accuracy. Additionally, Li et al., [83] assessed whether full-range spectra (400–4000 cm⁻¹) or only the fingerprint region (400–1800 cm⁻¹) contributed more to diagnostic accuracy. Their findings revealed less than a 2% difference in performance between the two methods, suggesting that the fingerprint region alone captures the most diagnostically informative content. This has practical implications, as focusing on the fingerprint region can streamline Raman spectral acquisition, reduce the computational load, and enhance clinical applicability without sacrificing accuracy. Detailed spectral analysis has revealed specific Raman bands that distinguish breast cancer subtypes and stages. Zeng et al., [85] identified characteristic peaks at 786, 1003, 1154, 1282, 1447, 1514, and 1580 cm⁻¹ with distinct assignments to cellular components such as cytosine, β-carotene, amide III (collagen), and lipids.Wang et al., [86] similarly reported prominent peaks at 950, 1004, 1154, 1280, 1445, 1514, 2517, and 2662 cm⁻¹ in their serum analysis.

### Photoacoustic spectroscopy with deep neural networks

**Fig. 7:**
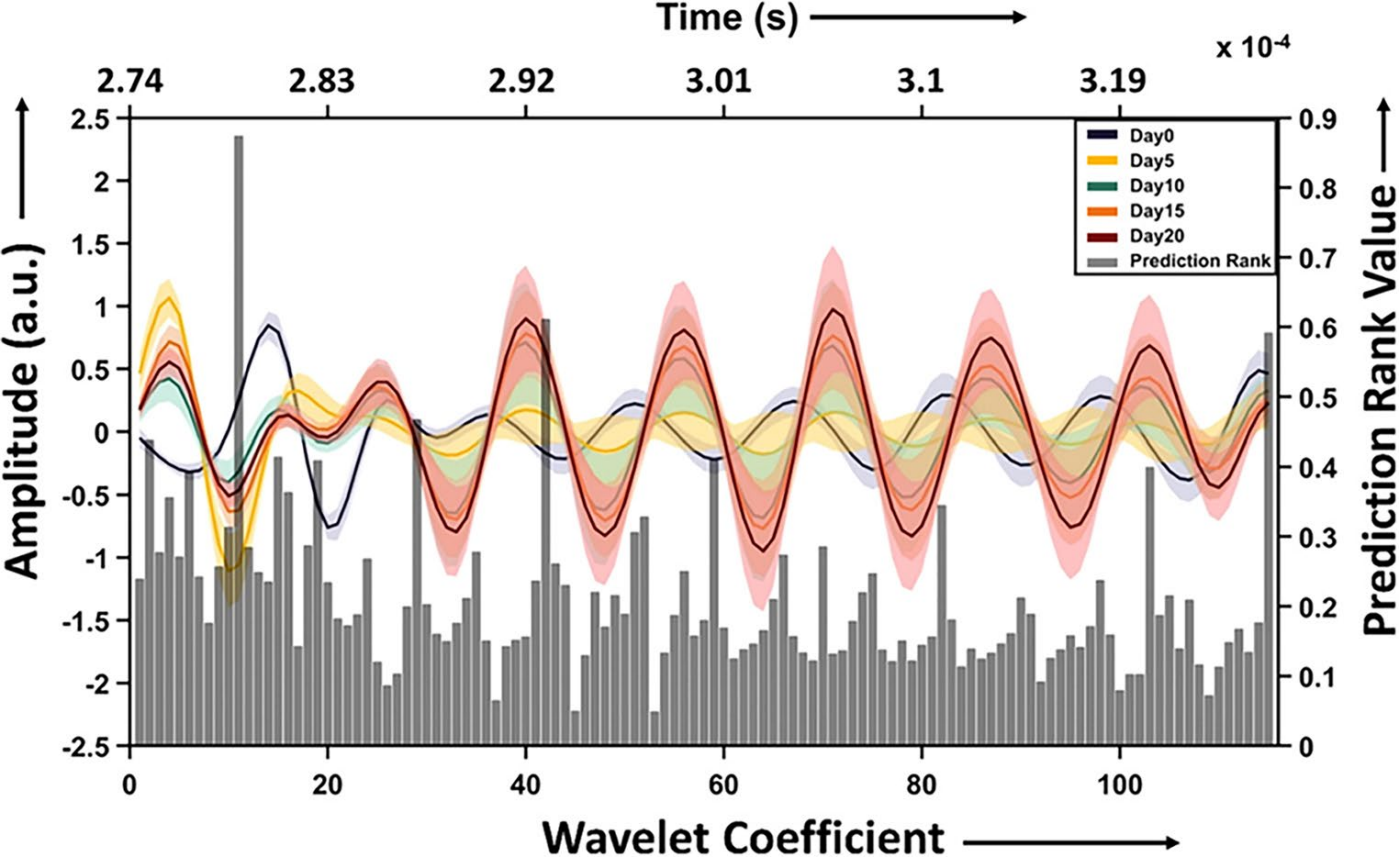
Mean photoacoustic spectra in the ROI. The bar graph represents the features after mRMR on the input data with a plot of wavelet coefficient versus prediction rank values. Figure adopted from, [60]

A critical component in applying ML to photoacoustic spectroscopy is the extraction and selection of relevant features from the acquired signals. Recent studies have employed wavelet transformation techniques to analyze time-domain photoacoustic signals. In particular, the continuous wavelet transform (CWT) has proven effective for converting these signals into time‒frequency domain representations, which can reveal subtle spectral changes linked to breast tumor development [87]. For feature selection, the minimum redundancy maximum relevance (mRMR) algorithm has gained prominence. This filter-based technique evaluates features via mutual information, balancing relevance and redundancy to discard extraneous variables that could compromise model accuracy [60, 87]. A notable study implemented mRMR to isolate the 20 most informative features from wavelet-transformed spectra, which were subsequently used to construct the input matrix for ML classification as shown in Fig. 7 [60].

Support vector machine (SVM) analysis has demonstrated considerable success in classifying photoacoustic spectral data for breast cancer detection. Studies have compared different SVM kernel functions, including the radial basis function (RBF), polynomial, and linear kernels, with SVM-RBF typically showing superior performance [15, 87]. This approach is particularly valuable because of its ability to handle high-dimensional data while maintaining classification accuracy. In a preclinical study using a mouse model, SVM-RBF achieved an overall accuracy of 94.5%, with a specificity of 100% and sensitivities ranging from 85% to 100% for different time points of tumor progression. Cross-validation testing of the SVM model revealed a mean accuracy of 97.5 ± 1.75%, demonstrating the robustness of this classification approach [87]. These findings highlight the potential of ML-enabled photoacoustic spectroscopy for noninvasive assessment of breast tumor progression. Recent advancements in DL have led to the development of sophisticated neural network architectures specifically designed for photoacoustic image analysis. A notable innovation is the integration of attention mechanisms with residual neural networks (ResNets) for improved feature extraction and classification performance [83].

Compared with conventional approaches, the PAUS-ResAM50 model, which combines a ResNet50 architecture with attention mechanisms for analyzing photoacoustic ultrasound (PA-US) images, has demonstrated superior diagnostic capabilities [83]. This model achieved impressive performance metrics in clinical validation, with an AUC of 0.917 (95% CI: 0.884–0.951), a sensitivity of 0.750, an accuracy of 0.854, and a specificity of 0.920 in the training set. Even in the testing set, the model maintained high performance, with an AUC of 0.870 (95% CI: 0.778–0.962). Li et al., [83] conducted comprehensive comparisons between different DL architectures applied to photoacoustic imaging. Compared with standard ResNet50 models without attention mechanisms, the attention-enhanced variants consistently demonstrated superior performance. Additionally, photoacoustic imaging models (PAUS-ResAM50) significantly outperformed those based solely on conventional ultrasound (BMUS-ResAM50), indicating the added diagnostic value provided by photoacoustic data. Statistical validation via the DeLong test confirmed the significance of these performance differences (*p* < 0.05), underscoring the value of both photoacoustic data and attention mechanisms in improving diagnostic accuracy. These findings suggest that DL approaches specifically tailored to PA imaging characteristics can extract more clinically relevant information than can standard architectures applied to conventional imaging modalities. A significant advancement in photoacoustic-based breast cancer diagnosis involves the incorporation of radiomic approaches that analyze features from both intratumoral and peritumoral regions. Research has shown that the combination of intratumoral and peritumoral photoacoustic imaging features yields superior diagnostic performance compared with the use of intratumoral features alone [88]. Huang et al., [88] investigated different peritumoral region sizes (2 mm, 5 mm, and 8 mm) and identified the 5 mm peritumoral region as providing optimal diagnostic information. The models incorporating both intratumoral and 5 mm peritumoral PA imaging features achieved AUCs of 0.924 (95% CI: 0.892–0.957) in the training sets and 0.873 (95% CI: 0.801–0.945) in the testing sets. This approach recognizes that tumor boundaries extend beyond visible margins, with the surrounding tissue environment providing valuable diagnostic information that can be captured through comprehensive radiomic analysis. To facilitate clinical application, researchers have developed nomogram models that integrate photoacoustic radiomic features with clinical risk factors. A comprehensive predictive model incorporating radiomic features from intratumoral and peritumoral 5 mm PA images with clinical risk factors achieved an AUC of 0.950 (95% CI: 0.925–0.975) in the training set and 0.899 (95% CI: 0.841–0.956) in the test set in the study by Huang et al., [88]. These integrated models demonstrate exceptional diagnostic capability in differentiating between benign and malignant breast nodules, potentially reducing the need for unnecessary biopsies. Decision curve analyses have further confirmed the significant clinical benefit of these nomograms for intervention in BI-RADS 3–5 breast nodules [88]. Comparative analyses by Gröhl et al., [87] between in vivo and ex vivo photoacoustic spectroscopy have revealed interesting insights. While ex vivo studies have shown slightly higher performance metrics (99% accuracy, 100% specificity, 98–100% sensitivity), in vivo applications still maintain impressive diagnostic capabilities (94.5% overall accuracy, 100% specificity, 85–100% sensitivity). The advantage of in vivo assessment lies in its ability to capture signals longitudinally from the same subjects during tumor progression, overcoming the heterogeneity issues associated with subject-to-subject variation in ex vivo studies.

A promising application of AI in photoacoustic imaging is its potential to serve as a diagnostic aid for radiologists. Li et al., [83] reported that integrating DL models with radiologist assessments can significantly enhance diagnostic accuracy. The PAUS-ResAM50 model, when used as an adjunctive tool, improved radiologists’ diagnostic specificity without reducing sensitivity, highlighting its value in clinical settings. This synergistic approach combines the interpretive expertise of radiologists with the computational power of AI, resulting in more accurate and consistent diagnoses. The integration of these technologies into clinical workflows has the potential to reduce reader variability and improve overall diagnostic performance, particularly for less experienced practitioners.

### Fluorescence spectroscopy with advanced machine learning models

Fluorescence spectroscopy has also gained significance due to advancements in ML and DL models. This technique enables the analysis of high-resolution, information-rich fluorescence images. In a study by To et al., [89], images are segmented into nonoverlapping patches and pretrained on models such as the ResNet architecture. ResNet50 is widely used because of its high accuracy and resilience against issues such as the vanishing gradient problem. Its skip connections are crucial for analyzing complex fluorescence images. Boosters such as XGBoost are employed because of their resistance to overfitting and their ability to handle imbalanced datasets. Additionally, Grad-CAM is used with DenseNet169, which connects each layer to every other layer, maximizing feature extraction, reducing redundancy, and increasing overall efficiency. While there have been significant advances in deep neural networks, classical ML techniques remain valuable alternatives when datasets are limited or when clinical explainability is essential. Support vector machines (SVMs) with radial basis function (RBF) kernels are used because they do not rely on raw pixel values; instead, they start with biologically handcrafted features transformed into a high-dimensional vector space for classification. This strategy allows models to capture both low-level visual cues and high-level structural patterns associated with molecular markers such as HER2, TP53, and BRCA2 [89].

### Diffuse optical spectroscopy and machine learning integration

Diffuse optical spectroscopy (DOS) has emerged as a promising noninvasive diagnostic technique capable of extracting rich biochemical and hemodynamic information via near-infrared light. Given the variability in patient datasets, selecting an ML model that is both accurate and robust becomes essential. Logistic regression, a classical ML technique, is exemplary in this regard. It is transparent and provides a clear, interpretable link between input features and predicted outcomes. It is also suitable for small datasets, where complex models such as SVMs and DNNs risk overfitting. Normalization and regularization further reduce this risk. Features such as hemoglobin, deoxyhemoglobin, and the tissue optical index demonstrated strong predictive power, making logistic regression an optimal model [90].

Furthermore, DOS has shown potential for integration into clinician decision-support tools [91]. It enables real-time assessment of tissue physiology via light absorption and scattering. To convert these spectral data into actionable diagnostic decisions, robust ML models are needed. A study by Nachabé et al., [91] provided a comprehensive evaluation of classifiers applied to physiologically derived broadband DOS data (500–1600 nm). Rather than applying ML techniques directly to raw spectral data, the study employed model-based fitting techniques to extract meaningful features such as hemoglobin, beta-carotene, lipids, collagen, water content, and light scattering parameters. For binary classification tasks, nonlinear SVMs (RBF kernels) and K-nearest neighbors (KNNs) outperform other models because of their flexibility and effectiveness with complex, overlapping biological data and their generalization performance on limited datasets. For multiclass settings, classification and regression trees (CARTs) outperform others because of their rule-based, interpretable structure, which handles subtle intertissue differences and achieves high accuracy despite repeated patterns. Linear models such as logistic regression and LDA, while transparent and simple, are limited by their inability to capture nonlinear relationships. Although promising, artificial neural networks (ANNs) pose a risk of overfitting with small datasets. Ultimately, the integration of diffuse optical spectroscopy with appropriate ML techniques can transform DOS from a data acquisition tool to a clinically relevant diagnostic aid. This approach is particularly valuable for real-time tissue characterization and treatment monitoring—areas where traditional techniques often lack specificity and sensitivity. A comparison of various AI techniques is presented in Table 2.

**Table 2:** Comparison of various AI techniques applied to spectral data

Model	Accuracy Range	Computational Cost	Hardware Requirements	Interpretability	References
CNN	84.5%-98.76%	High training cost (GPU hours), moderate inference time	GPU-accelerated systems required	Low inherent interpretability; requires Grad-CAM/SHAP	Bengio et al., [65], Aggarwal et al., [80], Alam et al., [66]
SVM (RBF)	82.14%-97.58%	Moderate training cost (O(nÂ²)), fast inference	CPU sufficient for small datasets	Moderate - Kernel visualizations & feature weights	Afrifa-Yamoah et al., [92]
Random Forest	94.47%-95%	Low-moderate training (parallelizable trees)	Multicore CPU beneficial	High - Feature importance metrics	Afrifa-Yamoah et al., [92]
Logistic Regression	89.7%-98.5%	Very low training cost	Basic CPU	High - Transparent coefficients	Afrifa-Yamoah et al., [92]
KNN	78.93%-90%	Low training (lazy learner), high inference	RAM-dependent for large datasets	Moderate - Similarity analysis	Afrifa-Yamoah et al., [92]
Decision Tree	80%-94%	Low training cost	Basic CPU	High - Rule-based structure	Afrifa-Yamoah et al., [92]

### Integration of multimodal data and spectral imaging

Modern breast cancer diagnostics are shifting away from single-modality point spectroscopy toward integrated, multidimensional approaches that offer superior diagnostic specificity. Multimodal fusion is the combining of functional data from spectroscopy with structural data from imaging (e.g., Ultrasound) which provides a more holistic view of the tumor microenvironment. For instance, models that integrate photoacoustic radiomics with clinical risk factors achieve significantly higher AUCs (0.950) than those using imaging alone. Techniques like fluorescence imaging and hyperspectral imaging (HSI) provide spatial context to biochemical signatures. These hypercubes require deep architecture like DenseNet169 to maximize feature extraction from the vast spatial-spectral overlap [83].

The advantages of multimodal fusion extend beyond improvements in diagnostic accuracy metrics such as AUC. Single-modality approaches are inherently limited by their inability to capture the full complexity of tumor biology for instance, spectroscopy provides high biochemical specificity but lacks spatial context, whereas imaging modalities offer structural information with limited molecular sensitivity [93]. Multimodal fusion addresses these limitations by integrating complementary data types, enabling simultaneous assessment of molecular composition, tissue architecture, and functional dynamics. The integration reduces biases, enhances robustness against noise and variability, and allows for a more improved characterization of tumor heterogeneity, including vascular, metabolic, and microenvironmental features. As a result, multimodal approaches enhance clinical interpretability and provide more reliable decision support compared to single-modality systems [94].

Nevertheless, there are still some challenges that should be overcome for efficient clinical implementation of multimodal fusion. For instance, it requires large, annotated data sets and advanced machine learning structures due to the high dimensional data and high computation cost required by multimodal fusion. In addition, due to the differences in temporal dynamics, data collection methods, and spatial resolution among different modalities, multimodal data registration and alignment remain challenging [95]. The dependability of the model might also be affected by inconsistency caused by the difference in the quality of data across different modalities. In terms of the clinical application of multimodal fusion technology, the use of multimodal fusion often involves higher costs, more complex equipment, and greater difficulty in incorporating the technology into current diagnostic protocols. Addressing these challenges will be essential to fully realize the potential of multimodal spectroscopic diagnostics in breast cancer care [96].

## Challenges and limitations

Optical spectroscopy has made great strides in cancer detection, but there are still a number of obstacles that prevent it from being widely used in clinical settings. These include the lack of defined acquisition and preprocessing procedures, low penetration depth in some spectroscopic modalities, variability in spectrum data due to tissue heterogeneity and apparatus variances, and difficulties in interpreting complicated spectral information. Furthermore, a lot of published research uses single-center validations or rather small datasets, which could restrict how broadly machine learning models can be applied. For clinical translation to be effective, these constraints must be addressed by standardized procedures, bigger multicenter datasets, and enhanced model interpretability. However, the integration of ML and DL with optical spectroscopy for breast cancer diagnosis faces significant technical and clinical hurdles. A primary challenge lies in the variability of spectral data quality, which arises from both biological and technical factors. Patient-specific variables such as age, hormonal status, and tissue heterogeneity introduce noise into spectral datasets, complicating the identification of consistent cancer-specific biomarkers [97, 98]. Technical inconsistencies, including differences in instrument calibration and environmental conditions during measurements, further degrade reproducibility. For example, LIBS-based studies comparing whole blood and serum samples revealed that minor variations in sample handling protocols significantly impact model accuracy, with whole blood yielding superior results (91.7% accuracy) compared with serum (89.7%) because of stronger emission profiles [97]. Similarly, Raman spectroscopy models trained on preprocessed data outperform those trained on raw or baseline spectra, underscoring the sensitivity of ML algorithms to data preprocessing steps [98]. These issues highlight the need for standardized acquisition protocols to ensure robustness across diverse clinical settings [99, 100].

Another critical limitation is the opaque decision-making processes of DL models, which hinder clinical trust and regulatory approval. While CNNs achieve high accuracy in subtype classification (e.g., 93% sensitivity for invasive cancer in Raman studies), their opaque nature obscures the biochemical rationale behind predictions [101, 102]. This lack of interpretability contrasts with simpler models such as SVMs, which provide transparent feature-weighting mechanisms but may sacrifice performance on complex datasets [100, 103]. Furthermore, validation frameworks remain inconsistent across studies, with some relying on single-institution datasets that fail to account for population diversity [104]. For example, Raman models classifying HER2-positive exosomes achieved 94% accuracy in controlled settings, but their generalizability to broader demographics remains unproven [35]. These validation gaps emphasize the need for multicenter trials and standardized reporting metrics to bridge the translational divide.

Clinical integration challenges extend beyond technical performance. Portable systems such as real-time FD-DOS probes demonstrate promising intraoperative applications (1.5 Hz processing speed) but face barriers in workflow compatibility [105]. Seamless integration with electronic health records, user-friendly interfaces for nontechnical staff, and compliance with medical device regulations remain unresolved [106]. Additionally, while diffuse optical tomography combined with ultrasound improves structural and functional assessments, most research has focused on binary classification (malignant vs. benign), neglecting the nuanced detection of molecular subtypes or treatment-resistant phenotypes [99, 107]. This narrow scope limits clinical utility, as personalized oncology demands granular prognostic insights. In addition to technical and clinical challenges, ethical issues present important limitations for the integration of machines and DL in optical spectroscopy for breast cancer. Algorithm bias can arise when models are trained on nonrepresentative datasets, potentially leading to reduced diagnostic accuracy in underrepresented populations and exacerbating health disparities [92, 108]. Data privacy is another major concern, as spectral and clinical data used for model training can contain sensitive patient information, increasing the risk of reidentification and misuse if not properly safeguarded (Afrifa‐Yamoah et al., 2025). The opaque nature of many DL models also complicates informed consent and accountability, as patients and clinicians may not fully understand how diagnostic decisions are made or who is responsible for errors. Furthermore, there is a risk of unethical practices in model development, such as data leakage or overfitting to specific datasets, which can artificially inflate reported accuracies and undermine clinical trust [109]. Addressing these ethical challenges is crucial to ensure that ML/DL-based spectroscopy tools are fair, transparent, and safe for widespread clinical use. Table 3 lists the advantages and disadvantages of using AI in optical spectroscopy for breast cancer diagnosis.

**Table 3:** Advantages and disadvantages of machine learning and deep learning techniques in cancer diagnosis: An enhanced analysis of the literature

Advantages of using ML and DL techniques	Disadvantages of using ML and DL techniques	References
Superior Pattern Recognition and Feature Extraction	High Data Requirements and Quality Dependency	Alam et al., [66], Afzalinia and Mirzaee [52], Aggarwal et al., [80], Afrifa-Yamoah et al., [92]
Enhanced Early Detection and Screening Efficiency	Significant Computational Resource Demands	Balasundaram et al., [34], Applegate et al., [105], Castorena et al., [69], Alam et al., [66]
Personalized Treatment Planning and Precision Medicine	Limited Interpretability and Transparency	Bhushan et al., [1], Al Assaad et al., [27], Carter et al., [109], Afrifa-Yamoah et al., [92]
Automation of Complex Diagnostic Processes	Susceptibility to Bias and Overfitting	Applegate et al., [105], Carter et al., [109], Afrifa-Yamoah et al., [92]
Scalability and High-Throughput Data Processing	Requirement for Specialized Technical Expertise	Alam et al., [66], Bhushan et al., [1], Castorena et al., [69], Afrifa-Yamoah et al., [92]
Improved Diagnostic Accuracy and Consistency	Variable Performance Across Populations and Contexts	Aggarwal et al., [80], Balasundaram et al., [34], Afrifa-Yamoah et al., [92], Carter et al., [109]
Integration of Multimodal Data Sources	Regulatory and Ethical Compliance Challenges	Al Assaad et al., [27], Bhushan et al., [1], Carter et al., [109], Afrifa-Yamoah et al., [92]

A significant concern in the current landscape of Al-enhanced optical spectroscopy is the overestimation of diagnostic performance, often termed “optimism bias”. While many pilot studies report near-perfect accuracies, these results are frequently deemed unreliable when subjected to rigorous external validation. This reliability gap typically arises from several technical and methodological pitfalls. High accuracies can be an artifact of the model overfitting specific noise patterns or experimental conditions rather than learning generalizable biological signals. This occurs when information from the test set inadvertently influences the training phase, often through improper cross-validation on small datasets [110]. Minor variations in instrument calibration, environmental light, and detector sensitivity can create unique spectral fingerprints. Models may achieve high accuracy by identifying these hardware-specific artifacts rather than the actual biochemical alterations in cancerous tissue. A large portion of existing research also relies on single-center validations with relatively small datasets. These models often fail to maintain performance when applied to data from different clinical environments or diverse patient populations (e.g., varying age or hormonal status), leading to a lack of generalizability [111]. Recent critiques have highlighted that many numerical ML approaches in optical analysis provide results that are not reproducible. For example, slight changes in sample handling such as the difference between whole blood and serum, can significantly shift model outcomes, suggesting that the high accuracy reported may be sensitive to specific laboratory protocols rather than robust pathology. To bridge this translational divide, it is essential to move beyond binary classification on clean datasets. Future work must prioritize independent external validation and the use of large, multicenter datasets to ensure that reported accuracies (e.g., those reaching 98%) are clinically robust and not merely artifacts of specific experimental conditions [112].

## Future direction

To fully harness the potential of machines and DL in optical spectroscopy for breast cancer applications, several key areas demand focused development. First and foremost is the creation of large, well-annotated, and standardized spectral datasets collected across multiple clinical centers, populations and instruments. These datasets facilitate model generalization, reduce bias, and enable reliable benchmarking of algorithms [113]. In addition to data expansion, there is a growing need for interpretable models. The integration of explainable AI (XAI) techniques such as SHapley additive exPlanations (SHAPs), attention mechanisms, and gradient-weighted class activation mapping (Grad-CAM) can improve the transparency of ML/DL systems by linking model predictions to specific spectral features, thereby fostering clinician trust and aiding regulatory review [98, 102]. Additionally, the combination of spectroscopy with other data modalities, such as imaging, genomics, and electronic health records, holds promise for obtaining a more holistic view of tumor biology. Multimodal fusion approaches using ensemble learning or advanced architectures such as transformers and graph neural networks (GNNs) can enhance diagnostic accuracy and subtype classification [103, 104, 114].

Ethical considerations, including patient privacy and data security, are increasingly being addressed through federated learning and privacy-preserving techniques [115, 116]. As regulatory frameworks evolve alongside these innovations, the goal is to ensure that ML/DL-enhanced optical spectroscopy becomes a reliable, safe, and accessible tool for early breast cancer detection and personalized care, ultimately improving patient outcomes and quality of life. Real-time deployment is another vital direction, especially for intraoperative and point-of-care settings. The development of lightweight, energy-efficient models suitable for edge computing will facilitate integration into portable spectroscopy devices, allowing bedside decision support even in resource-limited environments [117–120]. Moreover, the application of federated learning and lifelong learning models can allow continuous improvement of algorithms while preserving patient privacy and reducing the need for centralized data sharing. From a clinical perspective, long-term patient monitoring via spectral signatures and ML-based predictive modeling can enable personalized therapy adjustments and early detection of recurrence [121]. Finally, ethical and regulatory frameworks must evolve to keep pace with these technological advancements. This includes establishing standards for model validation, fairness across diverse populations, and seamless integration into clinical workflows. By addressing these interdisciplinary challenges, future research can ensure that ML and DL-enhanced optical spectroscopy are reliable and impactful tools for the early detection, diagnosis, and prognosis of breast cancer.

## Conclusion

Combining machine learning and deep learning with optical spectroscopy is an effective technique for detecting breast cancer. This approach provides new approaches for defining tissue in real time. Our comprehensive review reveals that these methods are quite effective in diagnosis, with CNN-based models achieving up to 97.58% accuracy in classifying tissue and 98.76% accuracy in identifying subtypes [79]. These methods maintain excellent sensitivity (90–100%) and specificity (85–95%) across several spectroscopic modalities. ML/DL-enhanced optical spectroscopy has the potential to change beyond what standard diagnostics can accomplish. These technologies allow researchers to detect alterations at the molecular level before they appear in conventional imaging. They can detect metabolic changes that occur before morphological changes. The ability to reliably classify breast cancer subtypes, including difficult subtypes such as triple-negative breast cancer (average AUC of 0.98), is a significant step toward personalized oncology [32]. Additionally, combining spectroscopic data with clinical characteristics in multimodal techniques outperforms single-modality systems. Despite decades of proof-of-concept research revealing the diagnostic potential of numerous spectroscopic methods, practical translation has been hampered by technical, clinical, and economic constraints. Data harmonization across institutions, model interpretability criteria for clinical adoption, and the need for thorough validation in various patient groups are all significant hurdles (Kelly et al., 2019). The lack of standardized protocols is a serious issue, and recent research has demonstrated that uniform data collection and processing methods are urgently needed. Small changes in how equipment is calibrated, the atmosphere, and sample handling can significantly affect how effectively a model performs [86]. This demonstrates how critical it is to establish solid quality control measures and operating procedures before using the model in a clinical context. Optical spectroscopic systems require more rigorous clinical validation frameworks, especially for continually learning algorithms that adapt to new data. The creation of standardized evaluation standards and independent testing platforms will be critical to guarantee consistent performance across clinical settings. In the future, the combination of ML/DL with optical spectroscopy holds enormous promise for improving breast cancer treatment. The combination of evolving AI architecture, improved hardware capabilities, and increased clinical acceptability predicts that these technologies will progress from research tools to clinical standards over the next decade. Overall, ML/DL-enhanced optical spectroscopy holds strong promise for improving early detection, personalized therapy, and clinical outcomes in breast cancer care. However, by addressing current limitations through standardization, explainability, and regulatory alignment, we can ensure that these powerful diagnostic tools fulfill their promise of improving patient outcomes, reducing unnecessary procedures, and advancing precision medicine in breast cancer care.

## Data Availability

Data may be available on request to corresponding author.

## Abbreviations

AI: Artificial Intelligence
ANN: Artificial Neural Networks
APSD: Area of Power Spectrum Density
BI-RADS 3–5: Breast Imaging-Reporting and Data System Categories 3 to 5
BILSTM: Bidirectional Long-Short-Term Memory Network
BMUS-ResAM50: B-mode Ultrasound with Residual Attention Module and ResNet50
BRCA2: Breast Cancer Gene 2
BT474: Breast Tumor 474
CART: Classification and Regression Trees
CI: Confidence Interval
CNN: Convolutional Neural Networks
CTCs: Circulating Tumor Cells
CWT: Continuous Wavelet Transform
DCIS: Ductal Carcinoma in Situ
DenseNet169: Densely Connected Convolutional Neural Network (169 layers)
DL: Deep Learning
DOS: Diffuse Optical Spectroscopy/Diffusive Optical Spectroscopy (duplicate)
ECM: Extracellular Matrix
ER: Estrogen Receptor
FAD: Flavin Adenine Dinucleotide
FDA: Fisher Discrimination Analysis
FD-DOS: Frequency-Domain Diffuse Optical Spectroscopy
GAN: Generative Adversarial Networks
Grad-CAM: Gradient-weighted Class Activation Mapping
GRU: Gated Recurrent Unit
GNN: Graph Neural Networks
HbO₂: Oxyhaemoglobin
HER2/HER-2: Human Epidermal Growth Factor Receptor 2
HHb: Deoxyhaemoglobin
Hs 578Bst: Human Breast Epithelial Cell Line 578Bst
k-NM: k-Nearest Neighbors
LDA: Linear Discriminant Analysis
LIBS-based: Laser-Induced Breakdown Spectroscopy-based
LSTM: Long Short-Term Memory
MCF-7: Michigan Cancer Foundation-7
MDA-MB-231: M.D. Anderson - Metastatic Breast 231
MDA-MB-468: M.D. Anderson - Metastatic Breast 468
miRNA: MicroRNA
ML: Machine Learning
metHb: Methemoglobin
mRMR: Minimum Redundancy Maximum Relevance
NAD: Nicotinamide Adenine Dinucleotide
NIR: Near Infrared
NNLM: Neural Network Language Model
nodes: Neurons
PA-US: Photoacoustic Ultrasound
PAS: Photoacoustic Spectroscopy
PAUS-ResAM50: Photoacoustic Imaging Models using ResNet50 and Attention Modules
PCA: Principal Component Analysis
PDT: Photodynamic Therapy
PLS-DA: Partial Least Squares Discriminant Analysis
RBF: Radial Basis Function
RF: Random Forest
RNN: Recurrent Neural Networks
ResNet50: Residual Neural Network with 50 Layers
ROI: Region of Interest
SERS: Surface-Enhanced Raman Scattering
SHAP: SHapley Additive exPlanations
SKBR3: Human Breast Cancer Cell Line SKBR3 (HER2-positive)
SSA: Sparrow Search Algorithm
StO₂: Oxygen Saturation
SVM/SVM-RBF: Support Vector Machine/Support Vector Machine - Radial Basis Function
tHb: Total Hemoglobin
TNBC: Triple-Negative Breast Cancer
TP53: Tumor Protein p53
VAEs: Variational Autoencoders
WM-DPAS: Wavelength-Modulated Differential Photoacoustic Spectroscopy
XAI: Explainable AI
